# Hæmorrhage before Delivery

**Published:** 1886-06

**Authors:** Odo Betz

**Affiliations:** Heilbron, Germany


### DANIEL'S

### Medical ★ Journal,

TEXAS

PUBLISHED MONTHLY AT

AUSTIN, TEXAS.

Vol. 1.] JUNE, 1886. [No. 12.

*"Scribimus indocti, doctique!"*

### Original Articles.

☞ CONTRIBUTED EXCLUSIVELY TO THIS JOURNAL. ☜

*The Articles in this Department are accepted, and published with the understanding that we are not responsible for, nor do we indorse the views and opinions of the writers, by so doing.*

### HÆMORRHAGE BEFORE DELIVERY.

*By Dr. Odo Betz.*

HEILBRON, GERMANY.

(For Daniel's Texas Medical Journal.)

THE writer was very much interested in a case of Hæmorrhage before Delivery reported by Dr. E. J. Dœring, of Chicago, in the November number of Daniel's Medical Journal. In view of the great importance of accidental hæmorrhage, and the high degree of mortality attending it, to both mother and child, I would like to call the attention of the readers of the Journal to a paper on this subject, read recently before the Berlin Gynæcological Society, by Dr. Winter, Assistant Physician to the Royal Lying-In-Hospital, entitled "Contributions to the Study of Hæmorrhage before Delivery," which throws considerable light on the etiology of this formidable complication of labor. In the three cases of accidental hæmorrhage reported by Dr. Winter, nephritis was found to exist as a complication; in two of them the nephritis was due to pregnancy and disappeared after confinement; in the other it continued, being chronic Bright's Disease. These three cases survived the hæmorrhage, but all the infants were still-born. Two of these cases occurred in the eighth month of pregnancy, the other in the seventh month. The symptoms indicating premature detachment of the normally situated placenta were alike in all the cases, consisting of severe pains in the abdomen, more or less external hæmorrhage and anæmia. Labor terminated spontaneously in two of the cases; in the third version was performed. In the absence of any of the usual causes of accidental hæmorrhage, Dr. Winter concluded that in these cases the etiology was clearly found in the existing nephritis.

In the discussion which followed, Dr. Lohbein stated that he remembered a case reported in the Berlin Klinische Wochenschrift in 1881, where hæmorrhage before delivery occured in a patient suffering from nephritis. He thought an explanation might be found in the increased arterial tension in nephritis, as shown by the very loud aortic sound and increased apex beat of the heart. Dr. Veit also mentioned two cases of nephritis followed by hæmorrhage before delivery from premature separation of the placenta. These observations should lead us to suspect nephritis whenever we meet with a case of accidental hæmorrhage, accompanied by the usual symptoms of severe pains in the abdomen, acute anæmia, external hæmorrhage, sudden increase in the size of the uterus, etc. On the other hand, whenever we recognize nephritis complicating pregnancy we should likewise prepare for a possible detachment of the normally situated placenta occuring before labor.

